# Periprosthetic Proximal Femoral Fractures: A Comprehensive Review of Epidemiology, Risk Factors, Classification and Management

**DOI:** 10.7759/cureus.100589

**Published:** 2026-01-01

**Authors:** Andrew Foster, Mohammad Waseem Beeharry

**Affiliations:** 1 Trauma and Orthopaedics, Royal Surrey NHS Foundation Trust, Surrey, GBR

**Keywords:** femoral, femur, fracture, implant, periprosthetic, periprosthetic proximal femoral fractures, ppff, vancouver classification

## Abstract

Periprosthetic proximal femoral fractures (PPFFs) represent an increasingly prevalent complication following hip arthroplasty, driven by global demographic shifts and rising arthroplasty rates. These fractures pose significant clinical and surgical challenges due to their association with implant instability, compromised bone quality, and increased patient morbidity and mortality. The Vancouver classification system remains the cornerstone for fracture classification and management decision-making, distinguishing fractures by location, implant stability, and bone stock quality.

This review outlines the epidemiology and risk factors associated with PPFFs, highlighting both patient-related factors, such as advanced age, female sex, osteoporosis, and comorbidities, and implant-related factors, including prosthesis type, fixation method, stem sizing, and surgical approach. Each fracture subtype within the Vancouver classification is discussed, with emphasis on appropriate surgical strategies ranging from conservative management to complex revision arthroplasty.

Recent innovations in surgical fixation, including angle-stable and double plating, offer improved biomechanical stability and enhanced union rates. Furthermore, advances in 3D printing and artificial intelligence hold promise for custom implant design and data-driven treatment planning. Novel techniques, such as transprosthetic drilling with internal cooling systems, may further address fixation challenges in complex cases.

As the burden of PPFFs continues to grow, a comprehensive understanding of risk stratification, evidence-based treatment, and emerging technologies is essential to improve outcomes and optimize healthcare resource utilization.

## Introduction and background

Periprosthetic proximal femoral fractures (PPFFs) refer to fractures occurring in the region of the femur surrounding or adjacent to a femoral implant, most commonly following primary or revision hip arthroplasty. These injuries typically involve the proximal femur and may compromise implant stability, bone stock, and overall hip function.

They are a growing clinical concern, largely due to the rising number of hip arthroplasty procedures being performed. As the population ages and the demand for joint replacements increases, the incidence of these fractures is expected to continue climbing. The incidence of periprosthetic femoral fractures (PFFs) following primary total hip replacement (THR) is currently estimated at 3.5%, with projections indicating an increase of approximately 4.6% per decade over the next 30 years. According to data from the UK National Joint Registry, PFFs are the third most common indication for revision surgery, and the number of revisions performed for this indication has nearly doubled since 2013 [1]. 

PFFs pose complex reconstructive challenges and are associated with significant patient morbidity, including prolonged hospital stays, impaired mobility, and increased mortality rates [2]. Timely diagnosis and a thorough understanding of patient- and implant-related risk factors are essential for optimizing treatment strategies and improving clinical outcomes. This article describes the epidemiology and risk factors of PPFFs, the diagnosis, management, and treatment rationale for these complex patients.

## Review

Epidemiology

The number of femoral fractures in the UK increased from approximately 64,000 in 2009 to 80,000 in 2022, representing a 25% rise over this period [3]. In contrast, the UK population grew by approximately 8.6% during the same timeframe [4]. This indicates an increase in both absolute and relative terms: not only are there more total cases (absolute increase), but the incidence of femoral fractures relative to the population size has also risen. Consequently, a greater proportion of individuals are now at risk of sustaining PPFFs, highlighting an escalating burden on healthcare services. Projections estimate that the incidence of PPFFs will continue to rise, with one study predicting at least a 4.6% increase per decade over the next 30 years [1].

Risk factors

PPFFs are multifactorial in origin, with risk factors broadly classified into two categories: patient-related and implant-related.

Patient-Related Factors

Patient-related risk factors for PPFFs are intrinsic characteristics of the individual that contribute to fracture risk independently of the surgical intervention, such as advanced age, low bone mineral density, female sex and hormonal changes as well as anatomical features like narrower femoral canals or thinner cortical bone.

Age is a well-established risk factor; individuals over 80 years have been shown to have a significantly increased risk of PPFFs, with a 2015 meta-analysis reporting a 4.2-fold greater incidence compared to younger counterparts [5]. This association is evident both postoperatively and intraoperatively, with studies demonstrating increased fracture susceptibility during surgery among older patients [6,7]. Notably, younger patients, particularly those under the age of 50, are also susceptible to intraoperative PPFFs (IOPPFFs), a risk that may be associated with narrower femoral canals requiring more forceful or prolonged rasping during implant insertion [8].

Bone quality plays a central role in fracture risk. Age-related decline in bone mineral density begins in early adulthood and continues progressively, with osteoporosis-characterized by severely reduced bone density-being strongly associated with PPFFs [9]. In women, the decline in circulating estradiol during the peri- and postmenopausal periods accelerates bone loss, contributing to higher fracture rates. Conversely, while testosterone levels decline with age in men, this is not independently predictive of reduced bone density [9]. Consequently, female sex is an independent risk factor for PPFFs, with women demonstrating nearly double the risk of intraoperative fractures and a 48% increased risk postoperatively [10]. Osteoporosis is present in approximately 60% of PPFF cases [11], highlighting the importance of skeletal health in this context.

The Dorr classification is commonly used to assess the bone quality and shape of the proximal femur and further informs risk stratification [12]. It categorizes the femur into three types based on cortical thickness and canal morphology: Type A exhibits thick, well-defined cortices; Type B shows moderate cortical thinning with a wider canal; and Type C is characterized by extensive cortical thinning and poor definition [12]. Dorr Type C femurs are particularly susceptible to PPFFs, especially in the context of uncemented prostheses, with reported fracture rates of 15.9% compared to 2.3% and 3.7% in Types A and B, respectively [8].

In addition to anatomical and skeletal factors, several comorbidities have also been associated with increased risk of PPFFs. These include cognitive impairment (e.g., dementia) and cerebrovascular events (CVA or TIA), and may contribute to impaired bone quality, falls, or postoperative complications [13].

Implant-Related Risk Factors

Implant-related risk factors for PPFFs include surgical variables such as whether the procedure is a primary or revision total hip replacement, the use of cemented versus uncemented prostheses, stem size and alignment, and the surgical approach employed.

Revision THRs are associated with a significantly higher risk of intraoperative PPFFs, with reported rates of 12.3% compared to 1.7% in primary procedures. Similarly, the five-year cumulative incidence of postoperative PPFFs (POPPFFs) is higher in revision cases (3.8%) than in primary THRs (0.8%) [14].

Another critical factor is the method of femoral component fixation. Uncemented stems are consistently associated with a higher risk of both intraoperative and postoperative fractures. Intraoperatively, Lamb et al. (2019) reported a 2.4-fold increased risk of PPFFs with uncemented implants [7]. Abdel et al. (2016) reported an even greater intraoperative risk, with uncemented stems associated with a 13.5-fold increase in fracture incidence compared to cemented stems. Furthermore, the long-term postoperative risk remains elevated, with a 20-year cumulative fracture probability of 7.7% for uncemented implants compared to 2.1% for cemented counterparts [14].

Stem sizing and alignment further impact fracture risk. Undersized stems increase the likelihood of stem subsidence by over 300%, while oversized stems elevate cortical stress by approximately 240%, though this latter finding approached but did not reach statistical significance (p=0.056). Additionally, varus stem alignment was found to be associated with increased cortical strain, whereas valgus positioning reduces primary implant stability [15].

The surgical approach also influences PPFF occurrence. Lygrisse et al. (2021) found that the direct anterior approach was associated with earlier PPFF onset (mean 12.5 days post-operatively) compared to posterior or lateral approaches (48.2 days), as well as higher rates of atraumatic fractures (78.3% vs 51.6%) [16]. Additionally, left-sided hip replacements carry an approximately 8% greater risk of intraoperative fractures, potentially related to surgeon handedness and operative ergonomics [6].

Diagnosis and management

The diagnosis of a postoperative periprosthetic femoral fracture (POPPFF) begins with a comprehensive clinical history, ideally obtained directly from the patient and supplemented by collateral information when available. Given that POPPFFs most commonly result from low-energy trauma, patients typically report a fall from standing height. However, it is important to note that a significant minority of cases (approximately 9%) may present without a clear history of trauma [17]. The clinical presentation often includes symptoms such as severe hip pain, impaired mobility, or complete inability to ambulate.

A focused physical examination should be conducted, during which tenderness on palpation and pain with hip movement are common findings. Additional signs may include localized swelling and shortening of the affected limb.

Radiographic evaluation is the initial investigative modality of choice for suspected PPFFs. This typically involves obtaining plain anteroposterior (AP) radiographs of the hip, as well as AP and lateral views of the entire femur. When plain radiographs are inconclusive or further characterization of the fracture pattern is required, computed tomography (CT) imaging may be used for more detailed assessment. These imaging studies facilitate fracture localization, assessment of prosthesis stability, and evaluation of bone quality. These factors are essential for classification using the Vancouver system as discussed below.

Vancouver Classification

The Vancouver classification categorizes PFFs based on fracture location, implant or prosthesis stability, and bone stock quality [18].

The first parameter is the fracture location, which is divided into three types: Type A fractures involve the trochanteric region and are further subclassified as Type AG (greater trochanter) and Type AL (lesser trochanter). Type B fractures occur around the femoral stem, while Type C fractures are located distal to the stem tip.

The second parameter is prosthesis stability, which is used to further subclassify Type B fractures. Type B1 fractures occur around a well-fixed femoral stem, indicating a stable implant.

The third parameter considers the quality of the surrounding bone (bone stock quality), which differentiates between Type B2 and B3 fractures. Type B2 fractures are characterized by a loose prosthesis with adequate bone stock, whereas Type B3 fractures involve both a loose implant and compromised bone quality. Table 1 summarizes the treatment options for each Vancouver type

**Table 1:** Vancouver Classification of Periprosthetic Femoral Fractures [19-21]

Vancouver Type	Description	Typical Treatment
A	Fracture of the greater (AG) or lesser (AL) trochanter	Typically conservative; ORIF (Open Reduction and Internal Fixation) may be used if displaced or symptomatic
B1	Fracture around the prosthesis; stem remains stable	ORIF is typical; conservative management can be considered in selective cases
B2	Fracture around the prosthesis; stem is loose, bone quality is good	Typically revision arthroplasty, ORIF may be considered depending on patient factors
B3	Fracture around the prosthesis; stem is loose, bone quality is poor	Typically revision with a long femoral stem; may also use cerclage wiring or structural grafts
C	Fracture is distal to the prosthesis	ORIF is standard; use of a plate that overlaps at least 50% of the stem length is recommended to ensure stability

Management of Vancouver Type A fractures: Type A periprosthetic fractures occur around the greater trochanter (AG) or lesser trochanter (AL). Although these fractures do not, in isolation, compromise femoral stem stability, their clinical significance relates to their potential effects on abductor function (AG) or calcar integrity (AL).

Type AG fractures are typically stable and may be treated non-operatively when displacement is less than 2 cm. The greater trochanter serves as an attachment site for the gluteus medius and minimus, vastus lateralis, and the short external rotators. It is subjected to substantial biomechanical forces, approximately twice body weight during ambulation and up to four times body weight during stair climbing. Consequently, disruption of this key attachment site may result in clinically significant morbidity, including abductor weakness, limp, non-union, and an increased risk of dislocation [19]. Conservative management generally involves restrictions on hip abduction and protected weight-bearing for up to 12 weeks. Surgical intervention may be indicated in cases of symptomatic non-union, persistent pain, instability, abductor insufficiency [20], or when displacement exceeds 2 cm [21] or 2.5 cm [20]. Operative fixation is typically performed with cerclage wiring, plates, or cables [19,20]. 

Type AL fractures are also usually managed non-operatively, except when a substantial calcar fragment threatens stem stability. In such cases, the fracture may be more appropriately classified as a Type B fracture, given the associated risk of prosthetic loosening [20]. Treatment depends on patient-specific factors and may include fixation with cerclage wiring with or without graft augmentation, or revision to a distal-fixation stem [21].

Management of Vancouver Type B1 fractures: Type B1 PFFs occur around a well-fixed femoral prosthesis, permitting displacement of the surrounding bone while maintaining implant stability; they represent the most common PPFF subtype, accounting for approximately 35% of cases and demonstrating favorable outcomes, including a 95% union rate, a 15% complication rate, and a 9% re-operation rate [22,23]. Management focuses on achieving stable fracture fixation while preserving the prosthesis, and both conservative and operative strategies have been explored.

Recent evidence suggests that non-operative management may be appropriate for a carefully selected subset of patients. Efird et al. (2023) reported no significant differences between operative and non-operative cohorts with respect to 1-year mortality, unplanned surgery, fracture union, or ambulatory status in patients with minimally displaced metaphyseal fractures without stem subsidence, although failures were more common in fractures located closer to the stem tip [23]. Crebert et al. (2025) similarly found no differences in mortality or weight-bearing status at 30 days, one year, or five years, supporting conservative treatment in frail or low-demand individuals [24].

Despite these encouraging findings, many surgeons continue to favor open reduction and internal fixation (ORIF), which remains the standard approach for displaced Type B1 fractures and is often selected even for minimally displaced cases due to its ability to restore alignment. A range of fixation methods is available, including strut allografts, cable or compression plates, combined constructs using cortical strut allografts, and locking plates. However, a comparative evaluation by Dehgan et al. (2014) of these constructs demonstrated that locking plates were associated with significantly higher rates of nonunion compared with cable plate or compression plate systems, raising concerns regarding their biomechanical and clinical reliability [25]. These inferior outcomes have been attributed to overly rigid constructs, stress risers at plate ends, premature loading, inadequate grafting, and the relative novelty of locking plate use, although further research is warranted to clarify these relationships [23,26].

To mitigate complications associated with locking plates, Larsen et al. (2012) recommended achieving anatomical fracture reduction, using adjunctive fixation such as cerclage wires to enhance stem stability, extending the plate from the supracondylar region to the major trochanter, and avoiding constructs that are excessively rigid and may impede bone healing [26,27].

Management of Vancouver Type B2 fractures: Type B2 PFFs occur around a loose femoral stem in the presence of preserved bone quality and structural integrity and, by definition, cannot be managed conservatively due to prosthetic instability. Traditionally, revision arthroplasty with a long-stem implant that bypasses the fracture site has been regarded as the standard of care, providing reliable fixation and mechanical stability while promoting fracture healing [20]. This approach is based on the principle that a stable, well-fixed stem is critical to allow immediate or early weightbearing and to prevent further mechanical failure.

Emerging evidence, however, has begun to challenge the notion that revision arthroplasty is universally required for all B2 fractures. A 2023 study comparing ORIF with revision arthroplasty reported no significant differences in infection rates, revision surgery, or re-operation rates at a mean follow-up of approximately 65 weeks, while ORIF was associated with significantly lower intraoperative blood loss [28]. These findings suggest that in selected patients, ORIF may provide adequate fracture stability while reducing the physiological burden of surgery. Supporting this, Di Martino et al. (2024) conducted a systematic review and meta-analysis of B2 and B3 fractures and reported that ORIF was associated with lower re-operation rates, reduced blood transfusion requirements, shorter hospital stays, and decreased operative time compared with revision arthroplasty [29].

Nevertheless, revision arthroplasty demonstrated superior union rates and long-term functional outcomes, highlighting the trade-off between minimally invasive fixation and definitive prosthetic stability [29]. Haider et al. (2021) additionally found no significant differences in radiographic or clinical outcomes between ORIF and revision arthroplasty, further supporting the potential role of ORIF in selected patient populations [30].

Biomechanically, ORIF relies on stable fixation of the fracture fragments to the existing stem without compromising implant integrity. This may be achieved through a combination of cerclage wiring, plate fixation, or structural allografts, depending on fracture pattern and bone quality. The success of ORIF is highly dependent on achieving anatomical reduction, adequate mechanical stability, and preservation of periosteal blood supply to support biological healing. Limitations of current evidence include heterogeneity in surgical technique, variability in fracture patterns, and surgeon preference for either ORIF or revision arthroplasty, which may introduce selection bias [29,30]. Furthermore, most studies have small sample sizes, limited follow-up, and lack randomized controlled designs, limiting the generalizability of their conclusions.

Taken together, these findings suggest that ORIF may represent a viable alternative to revision arthroplasty in carefully selected patients, particularly those with increased frailty, low to moderate functional demands, multiple comorbidities, or elevated anesthetic risk [31]. While revision arthroplasty remains the gold standard for ensuring long-term stem stability and predictable fracture union, ORIF may offer benefits in terms of operative efficiency, reduced perioperative morbidity, shorter hospitalization, and faster early recovery. Ultimately, patient-specific factors, fracture morphology, prosthesis type, and surgeon expertise must all be considered when determining the optimal treatment strategy. High-quality prospective studies, including randomized controlled trials and standardized reporting of outcomes, are required to define the precise role of ORIF in B2 fractures and to guide evidence-based decision-making in this complex clinical scenario.

Management of Vancouver Type B3 fractures*:* Type B3 fractures occur around the femoral prosthesis in the context of both a loose implant and compromised bone quality. The presence of poor bone stock significantly increases the complexity of treatment and limits fixation options. Current recommendations support the use of long-stem femoral implants in these cases, as the extended stem can bypass the fracture site and function similarly to an intramedullary nail, providing improved mechanical stability [32].

Adjunctive fixation techniques, such as cerclage wiring, are commonly employed to enhance construct stability, particularly given the reduced structural integrity of the surrounding bone. Christopher et al. (2016) demonstrated that the use of double-loop cerclage wires in cementless total hip replacements (THRs) increased peak torque resistance by 20% compared to constructs without cerclage support [33]. Importantly, cerclage wiring has not been associated with adverse outcomes related to fracture healing, including union rates, time to union, infection, re-fracture, or re-operation rates, indicating that it can be safely implemented without increasing the risk of complications [34].

When bone stock is insufficient to achieve stable prosthetic fixation, structural allografts can be employed to restore femoral geometry. Rasouli et al. (2012) describe two principal approaches for B3 fractures with poor bone stock: allograft-prosthesis composite (APC) and proximal femoral replacement (PFR) [35].

APC involves combining a structural allograft with a prosthetic femoral component to reconstruct the proximal femur. This technique is generally preferred in younger, more active patients, as it preserves bone stock and allows the potential for biological integration of the allograft over time. APC, however, requires a period of restricted weight-bearing to prevent mechanical failure and allograft fracture, and is associated with an increased risk of infection and graft resorption. Consequently, it is less suitable for frail or co-morbid patients, or for those who require early mobilization due to functional demands [35]. Long-term outcomes for APC are moderate; a 2009 study by Safir et al. reported a 15-year survivorship of 82.2%, with complications including nonunion at the graft-host junction, allograft fracture, and periprosthetic loosening [36].

PFR, in contrast, involves resecting the proximal femur and replacing it with a modular prosthetic component, effectively bypassing the need for biological fixation to compromised bone. PFR is typically reserved for frail, low-demand, or elderly patients, as it allows immediate or early weightbearing and rapid restoration of function. Despite these advantages, PFR carries a relatively high complication rate. The most commonly reported complication is dislocation, due to loss of native soft-tissue tension and abductor mechanism integrity, as well as wound-healing problems and distal re-fracture at the stem tip. Nevertheless, most patients achieve functional ambulation and remain largely pain-free at a mean follow-up of 3.2 years postoperatively [36].

The choice between APC and PFR must therefore balance patient age, functional demands, co-morbidities, infection risk, and expected rehabilitation capacity, with the ultimate goal of restoring mobility, minimizing complications, and maximizing long-term prosthetic survivorship.

Management of Vancouver Type C fractures: Vancouver Type C fractures are defined as those occurring distal to the femoral prosthesis. Despite their distal location, the proximity of the fracture to the prosthesis remains a critical consideration during fixation planning, due to the potential for secondary fractures or the development of stress risers [20]. The standard treatment for Type C fractures involves ORIF [20]. To mitigate the risk of stress risers, it is recommended that fixation plates overlap the prosthesis.

Kubiak et al. (2015) demonstrated that overlapping the plate with the prosthesis significantly increased the force required to cause construct failure by 273% compared to constructs with a 2 cm gap between the plate and prosthesis [37]. Similarly, Frobergh et al. (2012) reported that all patients who required reoperation following low-energy trauma resulting in stress-related refracture had plates overlapping less than 50% of the prosthesis length, supporting the recommendation that plates should span at least half the prosthesis to reduce the likelihood of stress-induced complications [38].

In a large retrospective study, Gausden et al. (2021) evaluated Type C fractures treated between 2004 and 2018 using lateral locking plates that bypassed the distal tip of the prosthesis in 98% of cases. They reported a 90-day mortality of 5% and a 2-year mortality of 31%, with a 13% reoperation rate and 10% incidence of non-union at two years [39]. These findings highlight that, although ORIF with appropriate plate overlap is generally effective for Type C fractures, careful attention to construct design and patient factors remains essential to minimize complications and optimize outcomes.

Emerging innovations and technological advances in PPFF management

While the future direction of PPFF management remains uncertain, current clinical trends and technological advancements offer valuable insight into potential trajectories of progress. With the global increase in hip arthroplasty procedures, the incidence of PPFFs is anticipated to rise correspondingly, thereby necessitating the development of innovative surgical techniques and implant technologies to address this growing clinical challenge [1].

Kösters et al. (2022) have identified several advancements in internal fixation strategies that hold promise for improving outcomes. Notably, angle-stable plates have demonstrated superior biomechanical stability and reduced contact area, which aid in preserving the periosteal blood supply. These plates are also associated with lower revision rates compared to conventional plating systems and can be implanted using minimally invasive approaches. Additionally, the use of double plating has emerged as a method to enhance primary stability, with evidence suggesting it may accelerate fracture union relative to single plating techniques [40].

Advances in technology are also influencing implant design and surgical planning. Li and Tian (2025) proposed the application of three-dimensional (3D) printing to manufacture patient-specific prostheses, customized according to individual anatomical and fracture characteristics. Moreover, they suggested that artificial intelligence (AI) may play a role in refining the Vancouver classification system, facilitating the development of data-driven algorithms to inform treatment decisions [41].

Although these innovations are still in the early stages of development, continued refinement and integration into clinical practice may substantially advance the management of complex PPFFs in the years to come.

## Conclusions

As the global population ages and the number of hip arthroplasties increases, the incidence of PPFFs is expected to rise significantly. Managing this growing burden will demand increased healthcare resources and more effective treatment strategies. The Vancouver classification remains a reliable framework for categorizing PPFFs and guiding intervention, though ongoing debate, particularly around Type B fractures, highlights the need for clearer, evidence-based management protocols.

Recent advances in surgical techniques and implant technologies, such as angle-stable and double plating, offer improved outcomes and reduced revision rates. Emerging tools like 3D printing and AI also promise to enhance implant customization and treatment planning. Together, these developments point to a future in which PPFF management is increasingly precise, patient-specific, and technologically advanced, thereby helping to mitigate the clinical and societal impact of these challenging fractures.
